# Development of hard X-ray photoelectron spectroscopy in liquid cells using optimized microfabricated silicon nitride membranes

**DOI:** 10.1107/S1600577524008865

**Published:** 2024-10-15

**Authors:** F. Capone, O. Muntada, J. C. Ramírez, M. J. Esplandiu, R. Dedryvère, A. Grimaud, B. Lassalle-Kaiser, D. Céolin, F. Pérez-Murano, J.-P. Rueff, Jordi Fraxedas

**Affiliations:** a Synchrotron SOLEIL L’Orme des Merisiers 91190Saint-Aubin France; b PHENIX Sorbonne Université, CNRS 75005Paris France; c Institute of Microelectronics of Barcelona (IMB-CNM) CSIC, Campus UAB, 08193Bellaterra, Barcelona, Spain; d Catalan Institute of Nanoscience and Nanotechnology (ICN2), CSIC and BIST, Campus UAB, 08193Bellaterra, Barcelona, Spain; e IPREM, CNRS, University of Pau & Pays Adour, E2S UPPA, 64000Pau, France; f Department of Chemistry, Merkert Chemistry Center Boston College Chestnut Hill MA02467 USA; g LCPMR Sorbonne Université, CNRS 75005Paris France; University College London, United Kingdom

**Keywords:** hard X-ray photoelectron spectroscopy, silicon nitride membranes, microfabrication, aqueous solutions, nanoparticles, beam damage, synchrotron radiation

## Abstract

We present first hard X-ray photoelectron spectroscopy (HAXPES) results of aqueous salt solutions and dispersions of gold nanoparticles in liquid cells equipped with specially designed microfabricated thin silicon nitride membranes, with thickness in the 15–25 nm range, mounted in a high-vacuum-compatible environment. The experiments have been performed at the HAXPES endstation of the GALAXIES beamline at the SOLEIL synchrotron radiation facility. The low-stress membranes are fabricated from 100 mm silicon wafers using standard lithography techniques. Platinum alignment marks are added to the chips hosting the membranes to facilitate the positioning of the X-ray beam on the membrane by detecting the corresponding photoemission lines. Two types of liquid cells have been used, a static one built on an Omicron-type sample holder with the liquid confined in the cell container, and a circulating liquid cell, in which the liquid can flow in order to mitigate the effects due to beam damage. We demonstrate that the membranes are mechanically robust and able to withstand 1 bar pressure difference between the liquid inside the cell and vacuum, and the intense synchrotron radiation beam during data acquisition. This opens up new opportunities for spectroscopic studies of liquids.

## Abstract 2

We have developed and successfully tested new environmental liquid cells for HAXPES studies using specially designed thin, low-stress silicon nitride membranes (15 to 25 nm thickness). The novel aspect is the fabrication of the membranes considering the geometry of the experimental setup, the dimensions of the beam and the use of platinum stripes to efficiently localize the membranes. The membranes are robust, able to withstand the 1 bar pressure difference between the liquid and vacuum, and the intense beam.

## 1. Introduction

X-ray photoelectron spectroscopy (XPS) is a widely used experimental technique that provides valuable information on the electronic structure of matter. Due to the electrical (negative) charge of the generated photoelectrons by the impinging X-rays, the inelastic mean free path (IMFP) of the photoelectrons, *i.e.* the mean distance travelled between consecutive inelastic collisions, lies in the low nm range in condensed matter (liquids and solids). This distance is a function of the kinetic energy (*K*) of the photoelectrons, which makes such a technique highly surface sensitive when using soft X-rays (typically below 2 keV) (Cardona & Ley, 1978 ▸; Hüfner, 2003 ▸). However, the IMFP can achieve values of tens of nm when hard X-rays are used (above 5 keV, approximately) in HAXPES (hard X-ray photoelectron spectroscopy) setups, opening up the possibility to characterize the chemical state of subsurface regions, and hundreds of nm for photon energies in the MeV range, a range mostly devoted to radiation-damage studies rather than for chemical characterization of solids (Powell & Jablonski, 2010 ▸; Shinotsuka *et al.*, 2017 ▸; Flores-Mancera *et al.*, 2020 ▸; Le & Nguyen-Truong, 2021 ▸).

On the way to the electron detector (analyser), the photoelectron intensity is attenuated depending on the gas load in the analysis chamber. In a gas or vapour, the IMFP decreases for increasing gas pressure *P* (IMFP ∼ *P*^−1^), with values in the cm range for about 10^−4^ mbar. Considering that electron analysers must be operated in high vacuum (HV)/ultrahigh vacuum (UHV) to guarantee a safe and long-lifetime operation of the detector, due to the use of high voltages and to the surface sensitivity of the technique, most of the laboratory commercial XPS systems operate in UHV conditions (<10^−8^ mbar) devoted to the characterization of solid surfaces.

However, from the early stages of the development of the technique in the 1970s, with the seminal work of Siegbahn (Siegbahn & Siegbahn, 1973 ▸; Siegbahn, 1974 ▸), there has been interest in the characterization of gases, liquids and their interfaces with solid surfaces. The combination of technical strategies to perform photoemission at higher pressures, initially in the low mbar range to allow studies around the triple point of water (6.12 mbar at 273 K), combined with the use of synchrotron radiation, have evolved to a status where local pressures of 1 bar or above can be achieved locally at the sample, an ideal situation to study catalytic processes in real conditions, while keeping a base pressure of a few mbar in the analysis chamber (Velasco-Vélez *et al.*, 2016 ▸; Amann *et al.*, 2019 ▸). At such high pressures, the IMFP becomes increasingly small (in the micrometre range), a distance at which it becomes critical for the sample’s surface to mechanically approach the entrance cone of the analyser in a controlled manner (Amann *et al.*, 2019 ▸; Schlueter *et al.*, 2019 ▸). At high pressures, XPS is termed ambient- or near-ambient-pressure photoemission spectroscopy (AP/NAP-PES or AP/NAP-XPS), and its extension to high-pressure/high-kinetic-energy regimes is normally referred to as AP-HAXPES.

Two main strategies have been adopted for performing photoemission in high-pressure environments, which can be classified generically as windowless and windowed. In the windowless case, the sample to be analysed (gas, liquid or solid) is in the chamber hosting the electron analyser (analysis chamber), without any physical separation between the sample and the analyser. In this case, the analyser is exposed to a pressure increase that must be controlled to protect it. Such protection is achieved by reducing the diameter of the analyser entrance cone and through successive differential pumping stages in the different sections of electron optics to progressively reduce the base pressure (Salmeron & Schlogl, 2008 ▸; Roy *et al.*, 2018 ▸). An alternative consists of attaching a cell to the analyser cone, thus separating the sample environment from the vacuum vessel; however, within this scenario, the analyser is exposed to the pressure increase produced in the interior of such a cell (Knudsen *et al.*, 2016 ▸).

Examples of windowless systems include the use of liquid jets, droplet trains and flat jets with dimensions in the tens of micrometres (Winter & Faubel, 2006 ▸; Pellegrin *et al.*, 2020 ▸), with base pressures in the analysis chamber up to 1 mbar. This is available especially on the HAXPES endstation of the GALAXIES beamline at Synchrotron SOLEIL using a liquid microjet setup (see *e.g.* Céolin *et al.*, 2017 ▸; Mosaferi *et al.*, 2024 ▸). Thin films of liquids (usually water, but also organic liquids) on solid surfaces with pressures in the 1–10 mbar range have been extensively explored with AP-XPS. Since the seminal works by Ogletree *et al.* (2002 ▸) and Denecke *et al.* (2002 ▸) using adapted commercial energy analysers, a host of works have been published in this field, which continues to be very active. A few selected examples highlighting the diversity of studies using AP-XPS encompass the surface enhancement of halides in aqueous solution interfaces (Ghosal *et al.*, 2005 ▸; Verdaguer *et al.*, 2008 ▸), the influence of surface rearrangements of bimetallic nanoparticles in real catalysts (Divins *et al.*, 2014 ▸), the chemical origin of the superhydro­phobic–superhydro­philic transition of TiO_2_ nanotubes (Macías-Montero *et al.*, 2017 ▸), or the reactivity of organic liquid electrolytes with metallic (Maibach *et al.*, 2019 ▸) or oxide battery electrodes (Dietrich *et al.*, 2020 ▸), among others.

Recent advances include the use of the dip-and-pull method and the use of Langmuir–Blodgett troughs. For the former, a solid surface (wafer or rod) is submerged in a liquid and XPS is performed in the meniscus formed in vacuum upon lift-off. This technique is appropriate for electrochemistry studies since the working and reference electrodes can be easily allocated in the liquid container (Favaro *et al.*, 2019 ▸; Favaro *et al.*, 2021 ▸; Källquist *et al.*, 2022 ▸; Capone *et al.*, 2024 ▸). For the latter, AP-XPS of Langmuir–Blodgett films allows the characterization of the electronic structure of the molecule/water interfaces *in situ*, for example as a function of the lateral compression (Hoek *et al.*, 2024 ▸).

The windowed strategy consists of physically separating the gas or liquid from the vacuum with thin solid membranes in dedicated environmental cells. Windowed cells are more able to accept a large variety of liquids incompatible with jet techniques such as viscous or corrosive liquids, while providing an ideal platform to investigate liquid/solid interfaces or electrochemistry by equipping the cell with electrodes. In this case, the liquid can be at pressures slightly above atmospheric pressure while keeping the analyser in HV/UHV (Kolmakov *et al.*, 2016 ▸; Weatherup, 2018 ▸). Such membranes must be photon and electron transparent and mechanically robust to withstand the pressure difference between the gas/liquid and vacuum, and the exposure to the intense synchrotron radiation beams. Because of the reduced IMFP of electrons in solids, the membranes must be thin, making HAXPES the technique of choice for membrane thickness typically above 10–15 nm (Kalha *et al.*, 2021 ▸). Graphene is an ideal material because of its 2D character (one atomic layer), its large in-plane mechanical strength due to the strong C–C bonding and its impermeability to gases and liquids. For such a system, the areas of the suspended membranes must be small (a few micrometres in diameter) to cope with the pressure difference and the membranes are usually supported in commercially available microfabricated grids (typically made of silicon nitride) and the vacuum tightness must be guaranteed. Several examples of XPS studies making use of graphene membranes can be found in the literature (Velasco-Vélez *et al.*, 2016 ▸; Kraus *et al.*, 2014 ▸; Velasco-Vélez *et al.*, 2015 ▸; Weatherup *et al.*, 2016 ▸). Other ultrathin (below 10 nm) free-standing membranes have also been prepared based on oxide materials such as graphene oxide (Kolmakov *et al.*, 2011 ▸) and TiO_2_ and Al_2_O_3_ (Lu *et al.*, 2020 ▸).

Silicon nitride is also a material of choice for nm-thick membranes owing to its mechanical stability and its fabrication using conventional lithography methods. As a result, membranes with a variety of dimensions are commercially available. Silicon nitride membranes have been mainly used for X-ray absorption (Velasco-Vélez *et al.*, 2014 ▸) and transmission experiments using microfabricated nanoreactors (van Ravenhorst *et al.*, 2018 ▸; Beheshti Askari *et al.*, 2020 ▸). In the case of nanoreactors, 20 nm-thick windows with diameters of about 8 µm have been fabricated. However, only a few HAXPES experiments have been reported using silicon nitride membranes (Masuda *et al.*, 2013 ▸; Tsunemi *et al.*, 2015 ▸; Suda *et al.*, 2021 ▸). The dependence of the IMPF for stoichiometric Si_3_N_4_ as a function of the electron kinetic energy in the 3–10 keV range, assuming a bulk density of 3.44 g cm^−3^ and using the TPP-2M model, is shown in Fig. S1 of the supporting information (red line). In this case, for 10 keV electrons, IMFP is 15.4 nm. In the 3–10 keV photon energy range, the calculated X-ray transmission for Si_3_N_4_ is nearly 1 (0.99557 and 0.99986 for 3 keV and 10 keV photons, respectively, for a 20 nm-thick membrane), *i.e.* almost completely transparent (Henke *et al.*, 1993 ▸). The IMFP in water for 7 keV electrons is about 19 nm (Shinotsuka *et al.*, 2017 ▸), so that the acquired HAXPES spectra correspond essentially to the liquid/membrane interface.

In this work, we present the first exploratory HAXPES results of aqueous solutions and dispersions of nanoparticles using specially designed microfabricated thin silicon nitride membranes (15–25 nm thickness) using both static liquid cells (SLCs) and circulating liquid cells (CLCs). The membranes, microfabricated on silicon chips and adapted to the size of the monochromatic synchrotron radiation beam, nominally 30 × 100 µm, are mechanically robust and withstand the 1 bar pressure difference between the liquid cell and vacuum, and the intense synchrotron radiation beam during data acquisition, with lifetimes well above 6 h when carefully handled and used. No endurance test has been performed to certify the lifetime of the membranes.

## 2. Experimental

### 2.1. HAXPES beamline and Scienta analyser

**Figure 1:**
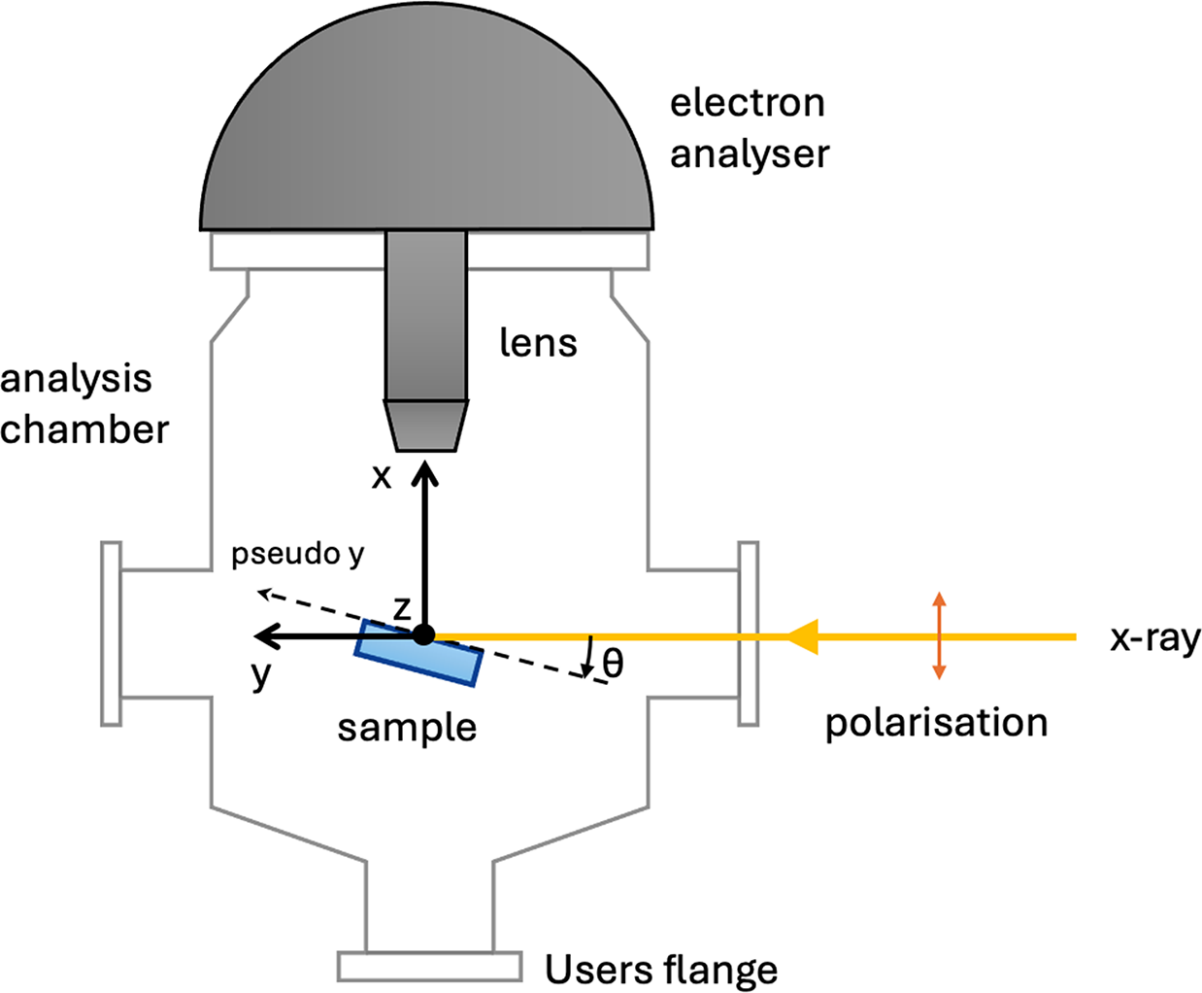
Sample geometry at the GALAXIES beamline (top view). The incident X-ray with horizontal linear polarization impinges on the sample at an angle θ. The emitted electrons are detected by the hemispherical electron analyser. The sample position can be adjusted along three directions denoted *x*, *y*, *z*, and the polar rotation θ. A pseudo axis (pseudo *y*) moves the sample along the surface.

A high-kinetic-energy Scienta EW 4000 hemispherical analyser installed on the HAXPES endstation of the GALAXIES beamline of the French national synchrotron facility SOLEIL was used for all the measurements (Céolin *et al.*, 2013 ▸; Rueff *et al.*, 2015 ▸). The photon polarization is horizontal linear and the energy resolution at 5 and 7 keV is 0.6 and 1 eV, respectively. The spectrometer lens axis (*x*) is collinear to the polarization. Its pass energy was set at 500 eV and we used 0.4 mm slit size, giving a theoretical resolution better than 400 meV. For the SLCs, we used the main motorized *xyz* manipulator of the HAXPES endstation, where the *y* axis corresponds to the beam axis and the *z* axis (height) is perpendicular to both the *x* and *y* axis. The manipulator also allows for motorized polar θ (*xy* plane) and azimuthal rotations (see Fig. 1 ▸). For the CLC, an *ad hoc* two-axis motorized manipulator was designed, providing both *x* and *z* motions. To protect the analyser from a sudden increase of pressure due to a leak in the membranes, security interlock switches were added to disable the applied voltages when the pressure is above 10^−4^ mbar.

### 2.2. Fabrication and characterization of silicon nitride membranes

Silicon nitride membranes were microfabricated in silicon chips and designed specifically for the HAXPES experiments at the GALAXIES beamline but can be easily adapted to other HAXPES experimental stations. SiN_*x*_ films have been deposited by low-pressure chemical vapour deposition (LPCVD). The use of an optimized LPCVD recipe instead of other common methods such as plasma enhanced chemical vapour deposition (PECVD) provides a more stoichiometric material (Si_3_N_4_) that results in enhanced mechanical properties. Stress measured on test wafers using the wafer curvature characterization method indicates a compressive stress around 200 MPa. As mentioned above, the thickness should be around 20 nm, to obtain sufficient photoelectron signal in the electron analyser. However, if the membrane is too thin, non-uniform or has high mechanical stress, there is a considerable risk that the membrane breaks down, which is a severe hindrance for a photoemission setup. Here, we have focused on the fabrication of membranes with nominal thickness of 15, 20 and 25 nm. Details of the whole fabrication process are presented in the supporting information (Fig. S2). In order to achieve an optimal balance between having a large enough membrane for the measurements, ensuring mechanical stability as well as conforming to the elliptical shape of the synchrotron radiation beams, we focus on rectangular membranes rather than the conventional approach of square membranes, since for rectangular membranes the strain depends mainly on the short dimension of the rectangle (Tabata *et al.*, 1989 ▸). This makes it possible to substantially increase the large dimension of the rectangle without affecting its mechanical response.

**Figure 2:**
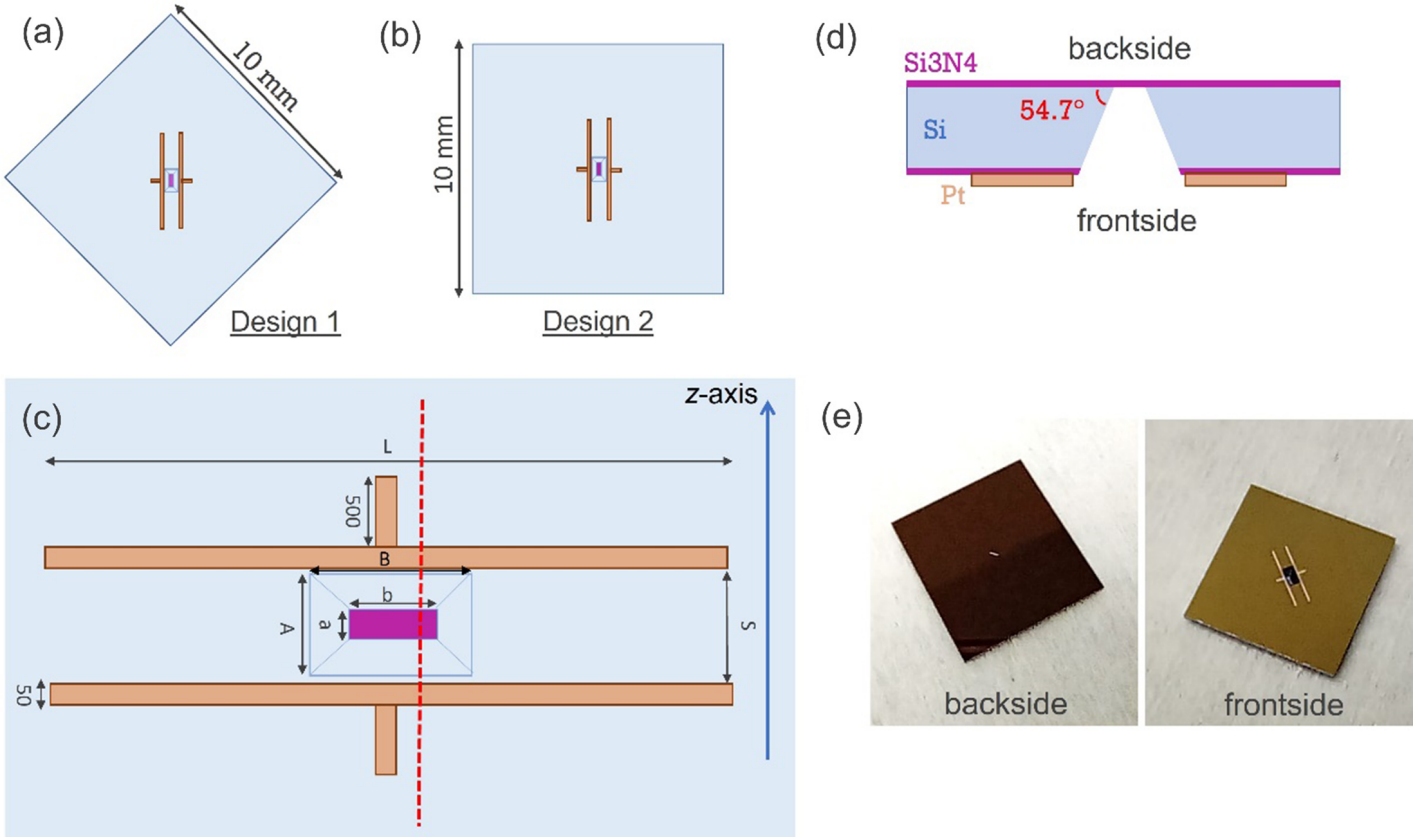
Design of the chips with a silicon nitride suspended membrane (not to scale). (*a*) Overview of the first chip design with the membrane placed diagonally with respect to the chip edges. (*b*) Overview of the second chip design, the membrane is orthogonal/parallel to the chip edges. (*c*) Detailed scheme of the platinum alignment marks (orange) and the silicon nitride membrane (purple). The dimensions of each parameter vary across designs and are detailed in Table 1 ▸. (*d*) Schematic cross section of the silicon chip (not to scale) and (*e*) images of the front side (right) and back side (left) of the chip.

Fig. 2 ▸ shows schemes and pictures of the two different geometries adapted to the SLCs used [design 1, Fig. 2 ▸(*a*)] and CLCs [design 2, Fig. 2 ▸(*b*)]. The dimensions of the silicon chips were set to 10 × 10 mm and the orientation of the membranes in the chips is such that the long dimension of the membrane must be contained in the *xy* plane (see Fig. 1 ▸). For the static cell, the membrane is aligned diagonally while for the circulating cell the membrane lies orthogonal/parallel to the chip edges because of the design of the cells (see Sections 2.3 and 2.4, respectively).

Platinum stripes as alignment marks [orange colour in Fig. 2 ▸(*c*)] are included to facilitate the positioning of the X-ray beam on the membrane [purple colour in Fig. 2 ▸(*c*)] by detecting the corresponding photoemission lines. Dimensions are detailed in Table 1 ▸ and vary across designs. A schematic cross section of the silicon chip [discontinuous red line in Fig. 2 ▸(*c*)] is shown in Fig. 2 ▸(*d*) and pictures of a SLC chip are shown in Fig. 2 ▸(*e*). The front and back sides of the membranes are in contact with the vacuum (facing incident beam) and liquid, respectively. Navigation to find the membrane with the photoemission signal is done as follows. A core level is selected (*e.g.* Pt 4*f*) and the cell is moved along the *z* direction until the two main stripes are localized. This sets the centre of the membrane along its short dimension. Then, one of the stripes is chosen, *e.g.* the top one. The cell is then moved downwards until the signal is lost. Then, the cell is moved along the chip surface (pseudo axis combining both *x* and *y* motions for the selected polar angle) until the signal from the small mark is identified. This position sets the centre of the membrane along its long dimension. With the acquired positions, the cell is sent to the beam location. Once there, the position is further optimized with the N 1*s* signal from SiN_*x*_.

Concerning the geometry of the membranes, two important points must be considered. First, the etching process of silicon imposes the morphology of the cavity, with an angle of 54.7° (see Fig. S3) and, second, the liquid must be in contact with the membrane on the flat part of the chip (back side) to guarantee good wettability and circumvent problems derived from surface tension (trapped air, bubbles *etc*.). These two conditions impose an incidence angle of the beam close to 45° in HAXPES experiments using the current chips since the direction of the beam (*y*) and the axis of the analyser (*x*) are perpendicular. If grazing-angle incidence is to be used, then the long dimension of the membranes should be substantially larger, which would make the membrane more fragile. Thus, our design must consider the fraction of the membrane that will be shadowed and thus not accessible to the beam. Illumination of such parts (close to the edges) would lead to a strong decrease of the photoemission signal of the lines of interest. This is illustrated in Fig. S3 (left). In consequence, on the one hand, the membrane needs to be large enough to ensure that the entire beam spot fits in its area. This allows us to maximize the beam–sample interactions as well as minimize undesired interactions with the rest of the chip. On the other hand, smaller membranes have higher mechanical stability. The relative sizes of the beam and the membrane are shown in Fig. S3 (right).

### 2.3. Static liquid cells (SLCs)

**Figure 3:**
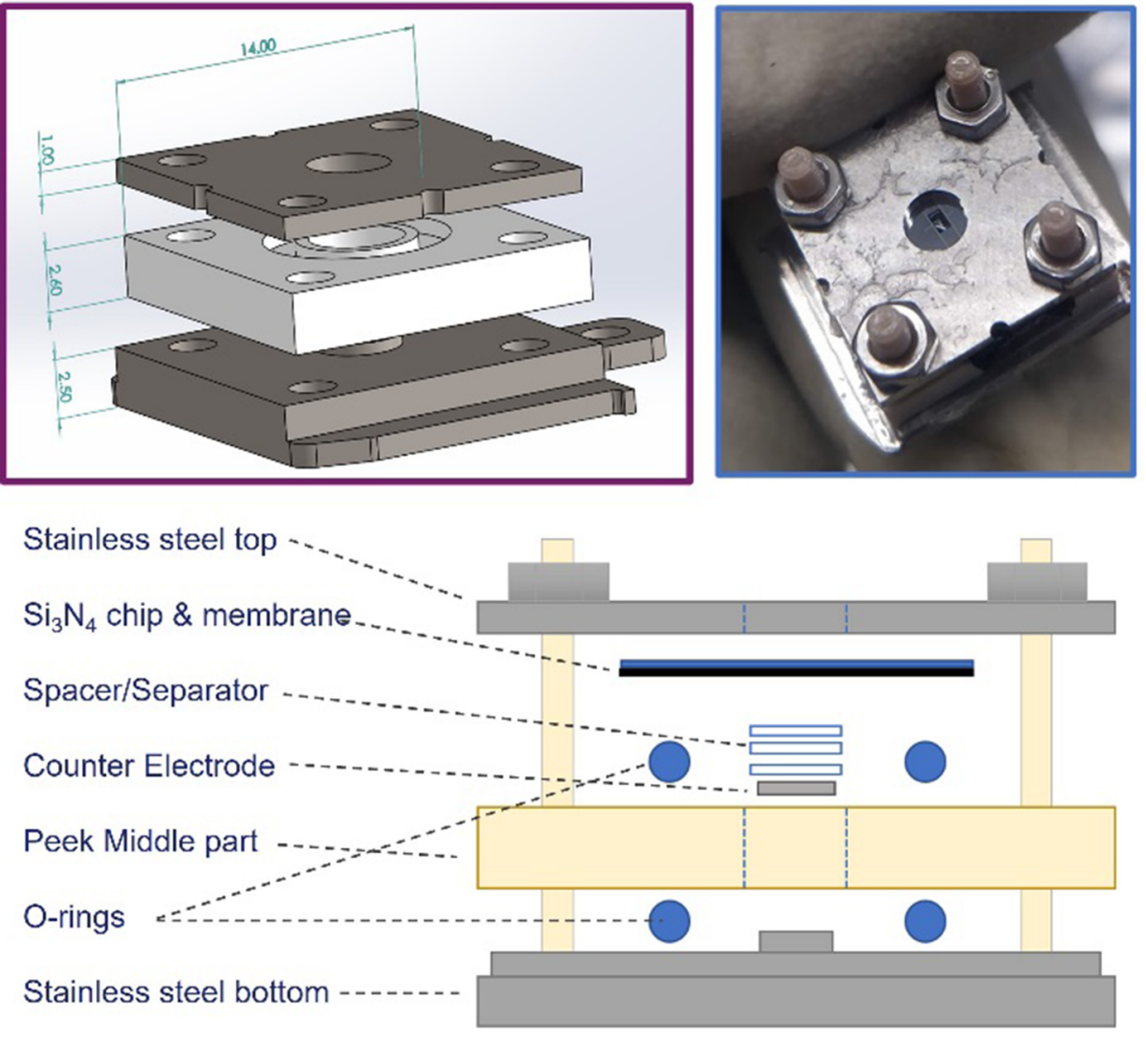
Schemes and picture of the static liquid cell. (Top left) Drawing of the three main pieces: Omicron-type stainless steel plate, PEEK spacer and stainless-steel cover piece. (Top right) Assembled cell with PEEK screws. (Bottom) Scheme of the parts used for electrochemical experiments.

The SLCs have been designed to fit on the sample manipulator of the analysis chamber, load-lock and transfer system of the HAXPES endstation, which uses a setup standardized for the Omicron sample plate. Schemes and a picture of a mounted cell are shown in Fig. 3 ▸. The top-left drawing shows the main components of the cell, which consists of three main pieces: an Omicron-type stainless steel sample holder plate, a spacer made from polyether ether ketone (PEEK) that defines the available volume of the liquid (about 30 µl) and holds the grooves for two Viton O-rings, and a 1 mm-thick stainless steel cover piece with a centred 3.5 mm hole. In the simplest configuration, the bottom plate and the top cover piece are in electrical contact with stainless steel screws and grounded through the sample manipulator. The top piece is also in contact with the platinum stripes. Thus, the liquid inside the container is in electrical contact with the holder plate which is grounded.

In a second configuration, the cell is adapted for electrochemistry experiments. In this case the bottom and top parts of the cell are electrically isolated using PEEK screws to avoid short-circuiting and control potential or current using a potentiostat (see top-right part of Fig. 3 ▸). The result is the sandwich-like design shown in the bottom part of Fig. 3 ▸. From the electrochemical point of view, this cell is envisioned to be used for studies on Li-ion batteries. Taking as an example a typical coin cell, the available diameter for the electrodes shrinks from 18 mm to 4 mm, but otherwise the cell assembly (in a glove box) and final product result highly resemble a normal coin cell. The much smaller diameter of the system as compared with the usual coin cell was limited by the maximum diameter attainable taking into consideration the size of commercially available O-rings and screws, and the need for parts thick enough in the PEEK and stainless steel to ensure mechanical stability. A sample parking specifically designed for this setup is available on GALAXIES on the manipulator of the analysis chamber that allows the user to freely apply potential separately to the top and bottom parts of the cell through an external device, as well as providing grounding to either part.

Once the liquid is introduced in the cell, the chips are fixed below the cover piece and the cell is mechanically sealed with four screws. A dynamometric screwdriver was used for tightening the four screws evenly to avoid damage both on the chip and on the membrane due to the confined liquid. Once in the fast-entry lock chamber, pumping must be performed slowly to avoid irreversible damage of the membrane caused by the sudden pressure difference.

### 2.4. Circulating liquid cells (CLCs)

**Figure 4:**
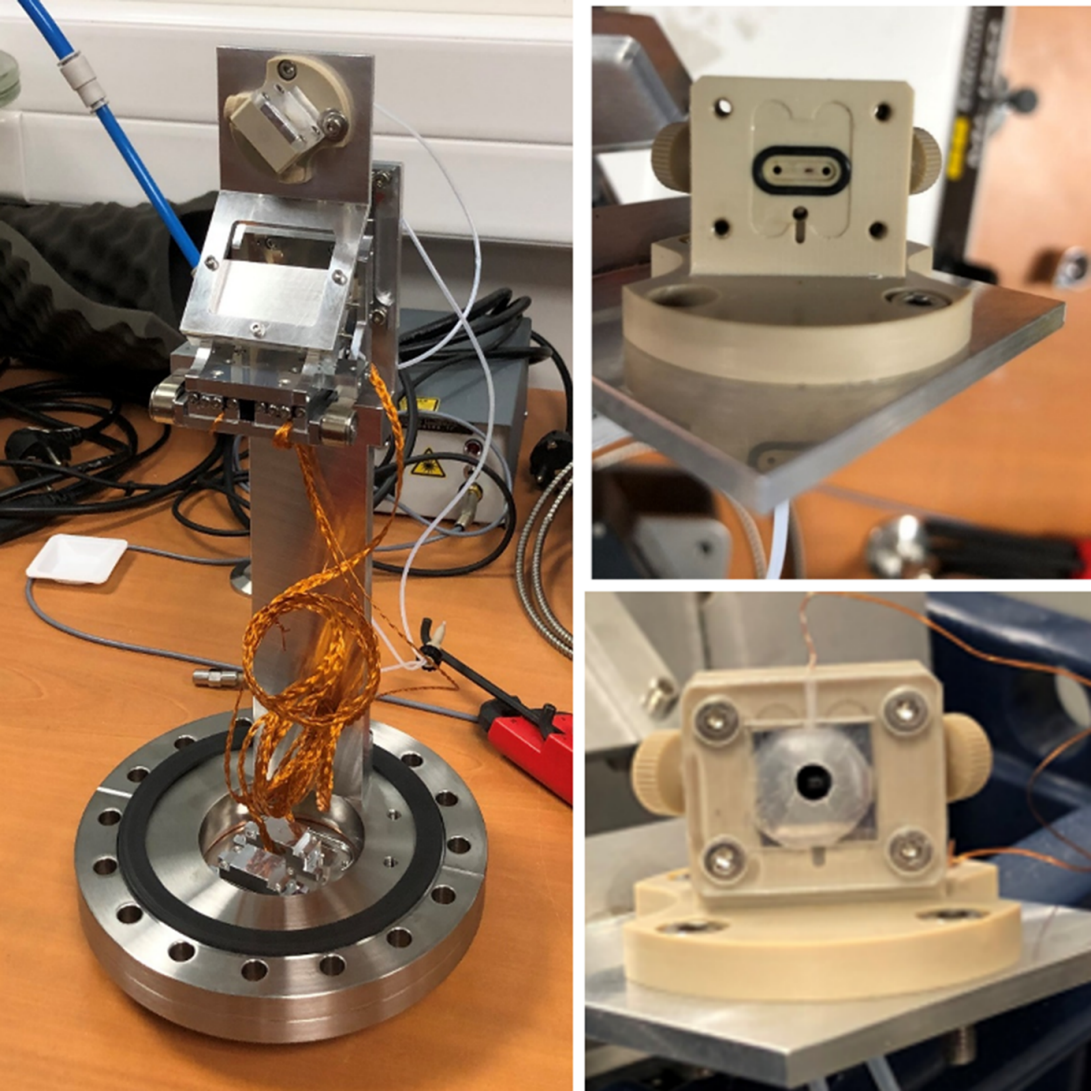
Pictures of the circulating liquid cell mounted in a DN100CF flange: (left) whole setup showing the motor stage and cabling and PTFE tubing (1/16 inch OD, 1 mm ID) and (right) detail of the PEEK cell without (top) and with (bottom) the chip with the membrane.

A first prototype of a liquid cell with circulating liquid used for the HAXPES experiments was based on an existing electrochemical cell at the LUCIA beamline at the SOLEIL synchrotron facility (Mendoza *et al.*, 2023 ▸). Fig. 4 ▸ (left) shows a picture of the cell, made from PEEK, mounted on a motor stage fixed to a DN100CF flange with sub-D feedthroughs. Two polytetra­fluoro­ethyl­ene (PTFE) tubes are shown which are used for the liquid inlet and outlet. The picture in the top right of the figure shows the cell body exhibiting the two circulation holes in the centre and the O-ring and a frame to insert the chip of the membrane. Finally, the bottom-right picture shows the chip installed in the cell fixed with a cover piece.

All membranes were checked with an optical microscope prior to installation in the liquid cell (see Fig. S4) and the liquid was circulated (cell in air) before installation in the analysis chamber to check for the stability of the membranes. The liquid flux used was 0.2 ml min^−1^. Charging was efficiently reduced by putting the front side of the chip (not in contact with the liquid) in contact with the metallic base of the motor stage through an aluminium foil piece. The pressure in the analysis chamber was in the 10^−6^ mbar range during measurements.

## 3. Results and discussion

### 3.1. 20 and 25 nm membranes using SLCs

**Figure 5:**
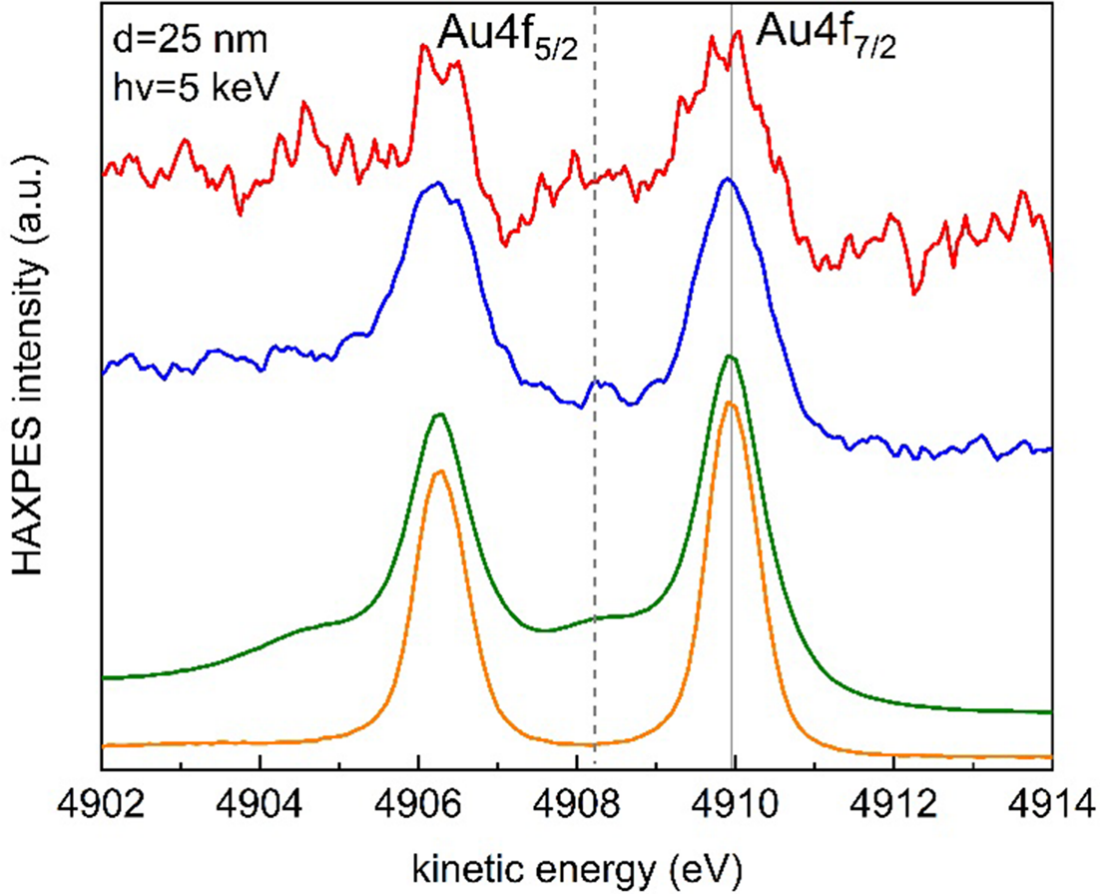
HAXPES spectra of the Au 4*f* region obtained with 5 keV photons of (orange) a 40 nm Au film and of (blue) a dispersion of Au NPs on a 25 nm-thick membrane in air and (red) a dispersion of Au NPs in MilliQ water in a static liquid cell. The green line corresponds to a previously measured Au film treated with oxygen plasma and measured with 1486.6 eV photons (Esplandiu *et al.*, 2018 ▸). The blue and red spectra have been shifted by +1.1 and −2.3 eV, respectively. The dashed line indicates the position of the oxidized Au 4*f*_7/2_ peaks.

First exploratory HAXPES experiments were performed with 25 nm-thick membranes decorated on the inner (back) side of the membrane with Au nanoparticles (NPs), of about 30 nm in diameter, using a SLC (see Fig. 3 ▸). Fig. 5 ▸ shows HAXPES spectra of the Au 4*f* region acquired with 5 keV photons. The blue and red lines correspond to the cell filled with air and a dispersion of Au NPs in MilliQ water, respectively, using two different membranes. The kinetic energies are normalized to the Au 4*f*_7/2_ line from a reference 40 nm Au thin film (orange line) deposited on a silicon wafer and obtained using a grounded Omicron-type sample holder (not the SLC). When the NPs are in contact with air in the SLC, the characteristic spin–orbit doublet is observed (blue line). In contact with water, extra lines shifted by 1.7 eV towards lower kinetic energies (dashed line in Fig. 5 ▸) reveal the oxidation of gold induced by the combined effect of the beam and water (radiolysis) (Weatherup *et al.*, 2018 ▸; Fraxedas *et al.*, 2019 ▸). A reference for the oxidation of gold is shown in the figure (green line), which corresponds to a previously measured Au film treated with oxygen plasma and measured with 1486.6 eV photons (Esplandiu *et al.*, 2018 ▸).

**Figure 6:**
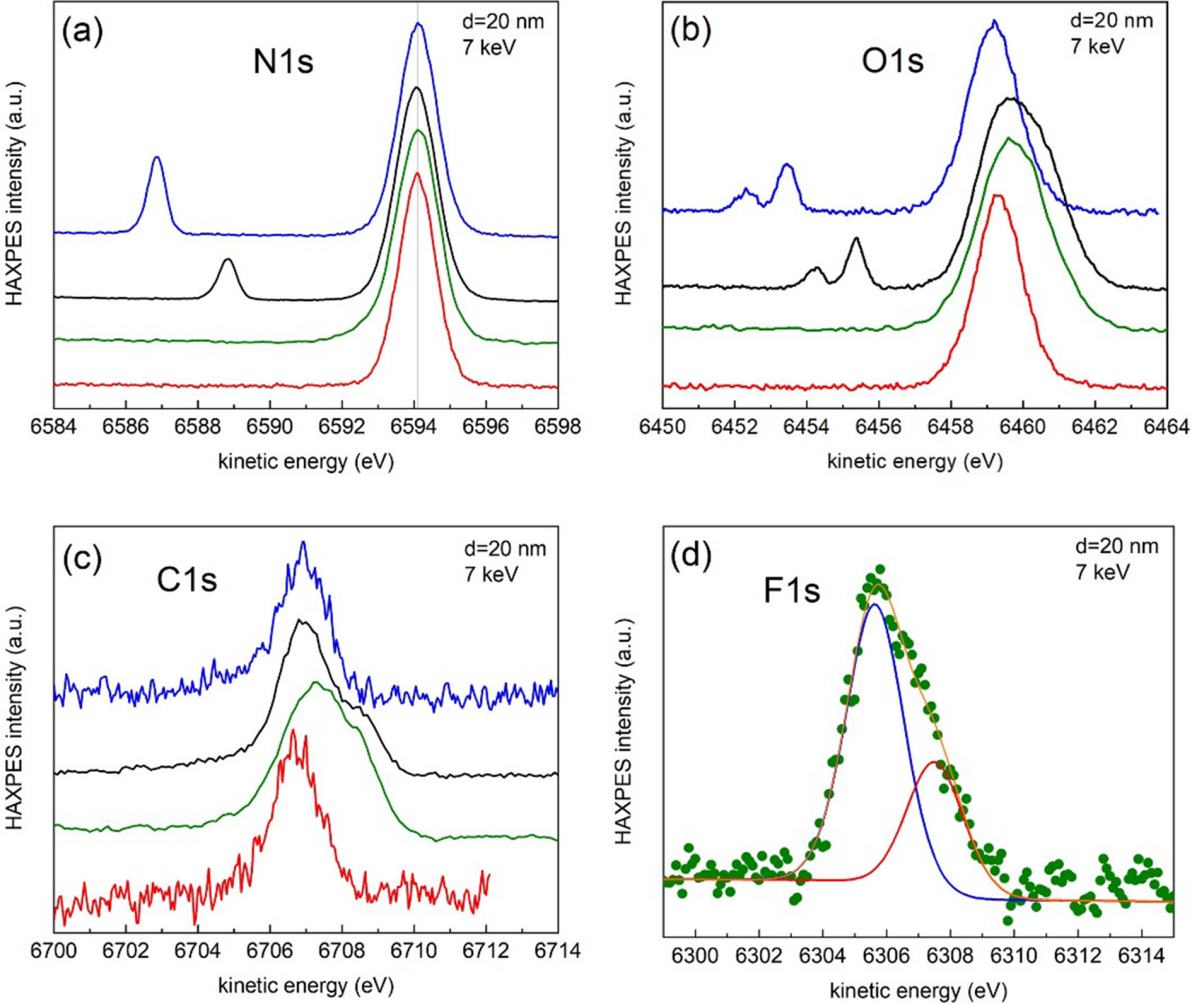
Normalized HAXPES spectra obtained with 7 keV photons of the (*a*) N 1*s*, (*b*) O 1*s*, (*c*) C 1*s* and (*d*) F 1*s* lines corresponding to four different membranes in the SLC filled with: air (blue lines), vacuum (red lines), with air and with a *ca* 5 nm-thick amorphous carbon coating deposited in the inner part of the membrane (black line) and with 60 µl of the LP57 electrolyte (1 *M* LiPF_6_ in EC:EMC 3:7) and with the *ca* 5 nm-thick carbon coating (green lines). The kinetic energies are referred to the N 1*s* line corresponding to the nitride of the sample coated with a carbon film [in air, black line in (*a*)]. The heights of all spectra are normalized to their corresponding maxima. The blue, red and green spectra have been shifted by −1.25, −0.9 and −1.5 eV, respectively. A least-squares fit using the *CasaXPS* software (Walton *et al.*, 2010 ▸) after a Shirley-type background subtraction using a combination of Gaussian (70%) and Lorentzian (30%) functions under the constraint of identical full width at half-maximum (FWHM) for all components is shown in (*d*). The envelope from the fit is represented by the orange line, which closely follows the experimental data.

Further experiments performed with 20 nm-thick membranes in the SLC are described next. Fig. 6 ▸ shows the N 1*s* (*a*), O 1*s* (*b*), C 1*s* (*c*) and F 1*s* (*d*) lines, corresponding to four different membranes in the SLC filled with: air (blue lines), vacuum (red lines), with air, but with a *ca* 5 nm-thick amorphous carbon coating deposited in the inner part (back side) of the membrane (black line) and with 60 µl of a LP57 classical Li-ion battery electrolyte (1 *M* LiPF_6_ in EC:EMC 3:7) and with the *ca* 5 nm-thick carbon coating (green line). The kinetic energies are normalized to the N 1*s* line corresponding to the nitride of the membrane coated with a carbon film [in air, black line in Fig. 6 ▸(*a*)], since such a coating works as an electrode that is to be grounded together with the analyser during measurements. We observe the presence of smaller peaks in the N 1*s* region when the SLC is filled with air exhibiting lower kinetic energies, which is assigned to molecular nitro­gen (gas phase). Note that the position of such a peak is different depending on whether the inner part of the membrane is coated (black line) or not (blue line) with carbon, and the shift with respect to the main nitride feature decreases when the membrane is grounded. This is thus an indication of the charging in the cell. The same effect is observed for the O 1*s* line in Fig. 6 ▸(*b*), where molecular oxygen is clearly identified by its doublet with 2/1 ratio arising from the paramagnetic character of the molecule. The energy difference between the main N 1*s* (nitride) and gas lines is 5.3 and 7.2 eV, when the inner part of the membrane is coated or uncoated with a thin carbon film, respectively. The shift is thus 1.9 eV, the same as for the O_2_ gas lines (Shah *et al.*, 2019 ▸; Avval *et al.*, 2019 ▸). The O 1*s* and C 1*s* spectra corresponding to the membranes capped with carbon coating [black and green lines in Fig. 6 ▸(*b*) and 6 ▸(*c*), respectively] show extra contributions towards higher kinetic energies that are ascribed to the carbon coating while the lower kinetic energy lines arise from surface contamination.

Fig. 6 ▸(*d*) shows the F 1*s* line, which corresponds to the used electrolyte. The peak can be fitted with two components that can be ascribed to the fluorine in the dissolved LiPF_6_ salt (highest blue component on the left) and to LiF (red component), a common reaction product of this salt found at the interfaces in these systems. The kinetic energy difference between the two signals is in agreement with what is commonly observed in Li-ion batteries (Dedryvère *et al.*, 2005 ▸).

### 3.2. 15 nm membranes using CLCs

**Figure 7:**
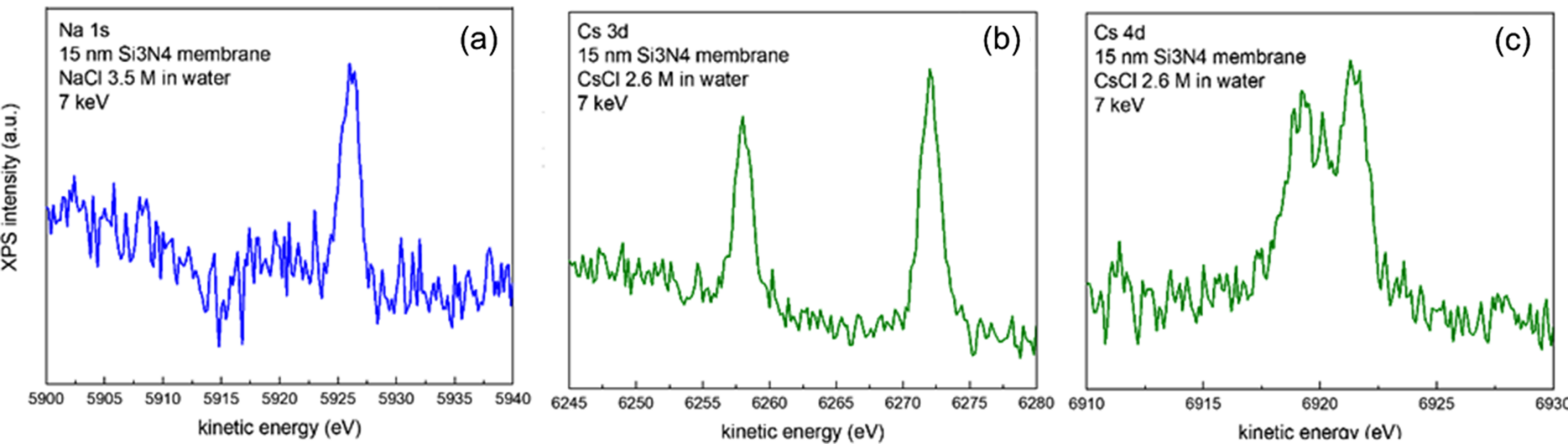
HAXPES spectra of: (*a*) the Na 1*s* region of a 3.5 *M* NaCl aqueous solution and (*b*) Cs 3*d* and (*c*) Cs 4*d* lines of a 2.6 *M* aqueous solution of CsCl obtained with 7 keV photons and using 15 nm membranes.

First experiments using the CLC and 15 nm-thick membranes were performed with concentrated aqueous solutions of NaCl (3.5 *M*) and CsCl (2.6 *M*) with the liquids circulating with a flux of 0.2 ml min^−1^. The Na 1*s* (*a*), Cs 3*d* (*b*) and Cs 4*d* (*c*) lines are depicted in Fig. 7 ▸. The selection of the photon energy is not critical, but from Fig. S1 we observe that considering 15 nm-thick membranes, IMFP = 15 nm is achieved for about 10 keV photons and that the maximum of the estimated peak intensity using the *SESSA* software (Werner *et al.*, 2021 ▸) (for the particular case of the Cd 3*d*_5/2_ line) is larger for 5 keV photons. However, for 5 keV photons, IMFP decreases to 8.6 nm. For this reason, we have chosen 7 keV, with an estimated IMFP of 11.4 nm. Our results are to be compared with previous similar experiments performed in a laboratory XPS system using environmental cells with commercial microchips with 5 nm thick and 30 × 30 µm wide silicon nitride membranes and a conventional Al *K*α excitation source in normal emission (Endo *et al.*, 2019 ▸).

## 4. Conclusions

We have developed new environmental liquid cells for HAXPES using specially designed thin, low-stress silicon nitride membranes (15 to 25 nm thickness). The cells have been successfully tested with aqueous solutions of salts, dispersions of gold nanoparticles and in electrochemical experiments. The nominal dimensions of the beam (30 × 100 µm) set the smaller dimensions of rectangular membranes. The geometry of the HAXPES endstation of the SOLEIL facility (horizontal polarization parallel to the analyser axis) together with the intrinsic 54.7° angle arising from the etching of silicon for the fabrication of the membranes impose an incidence angle of about 45°, excluding grazing incidence because of the large footprint of the beam. Optimizing such dimensions, we have produced nominally 15 nm-thick membranes that can be safely used for HAXPES experiments of liquids (and gases). When the membranes are inspected prior to assembly in the cells and carefully handled, pumping from atmospheric pressure is performed slowly enough and unnecessary exposure to the beam is avoided (beam shutter closed when data are not acquired and efficient localization of the membrane with the platinum guiding lines), then the lifetime of the membranes is beyond the time scale of the performed experiment in our tests, which amounts to more than 6 h. We believe that our results open the way to regular HAXPES studies of liquids under circulation and potentially to other techniques using synchrotron radiation.

## 5. Related literature

The following reference, not cited in the main body of the paper, has been cited in the supporting information: Bowden *et al.* (1998 ▸).

## Supplementary Material

Supporting information. DOI: 10.1107/S1600577524008865/ing5004sup1.pdf

## Figures and Tables

**Table 1:** Dimensions of the components of the chips for the two designs The estimated mean force due to the pressure difference (10^5^ Pa) for a 50 × 600 µm membrane is about 3 µN.

	Size (µm)	
Parameter	Design 1	Design 2
Beam alignment marks length (L)	3500	4000
Beam alignment marks separation (S)	820	900
Back side membrane width (A)	800	770–800
Front side membrane width (a)	∼90	∼20–50
Back side membrane length (B)	1250	1350
Back side membrane length (b)	∼530	∼600
