# Supplementary material for: Development of hard X-ray photoelectron spectroscopy in liquid cells using optimized microfabricated silicon nitride membranes
Source: J Synchrotron Radiat. 2024 Oct 15;31(Pt 6):1505–13. doi: 10.1107/S1600577524008865 (PMC11542648; doi:10.1107/S1600577524008865)
Supplement: Supplementary file 1 [file s-31-01505-sup1.pdf]

JOURNAL OF SYNCHROTRON RADIATION

Volume 32 (2025)

Supporting information for article:

Development of hard X-ray photoelectron spectroscopy in liquid cells using optimized microfabricated silicon nitride membranes

F. Capone, O. Muntada, J. C. Ramirez, M. J. Esplandiu, R. Dedryvere, A. Grimaud, B. Lassalle-Kaiser, D. Ceolin, F. Perez-Murano, J.-P. Rueff and Jordi Fraxedas

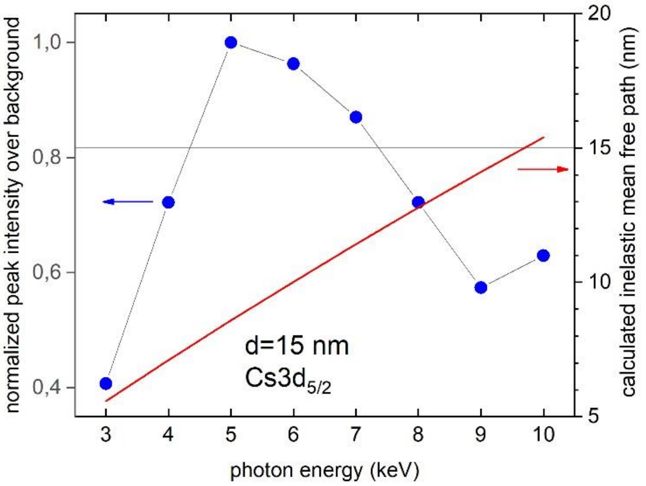

Fig S1. Calculated normalized peak intensity of the Cd3d$_{5/2}$ line over the background level in the 3 to 10 keV photon energy range using the SESSA software (blue dots) [1] compared to the simulated inelastic mean free path of silicon nitride in the same energy range (continuous red line), assuming a 3.44 gr cm-3 density and using the TPP-2M model.

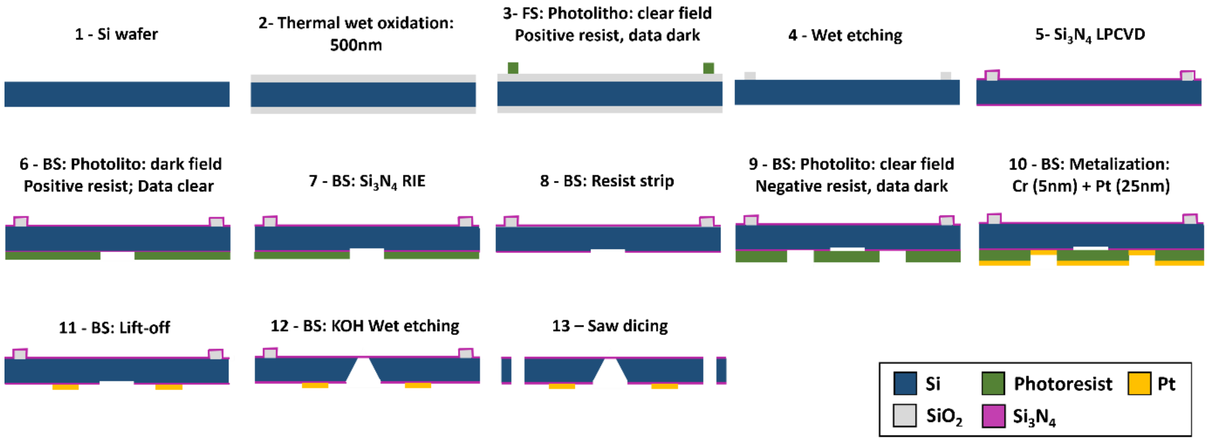

Fig S2. Fabrication process flow for the membrane-containing chips. Steps 1- 4 correspond to the fabrication of alignment and sawing marks by silicon oxide grow, photolithography and wet etching. Step 5 is the deposition of silicon nitride that will constitute the membrane material and also serves as a mask during wet etching in step 12. Steps 6-8 define by means of photolithography and dry etching the front side aperture of the cavity to obtain the suspended membrane. Steps 9 – 11 illustrate the fabrication of the Pt alignment marks for beam positioning. Step 12 is the anisotropic etching of the bulk silicon that frees the membrane. Finally, in step 13 the wafer is diced into chips of the size required to fit into the liquid cell.

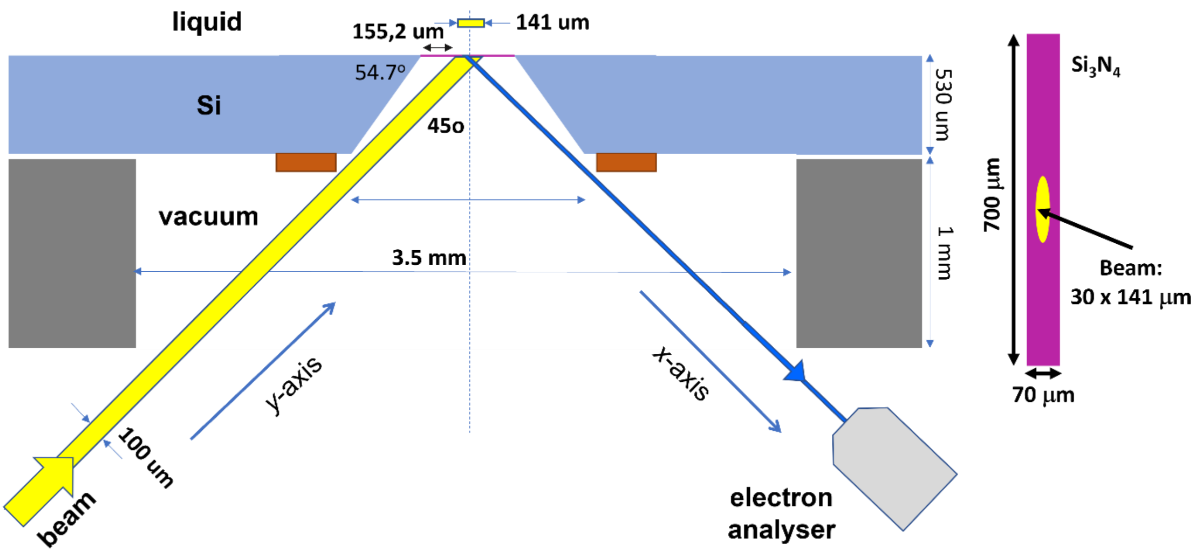

Fig S3. (left) Cross-section of the interaction between the beam and the chip (not to scale) and (right) projected image of the beam in comparison to the membrane. Note that the nominal size of the beam is 30 x 100 µm^2 but since it contacts the membrane at 45° the projected beam on the membrane is 30 x 141 µm^2.

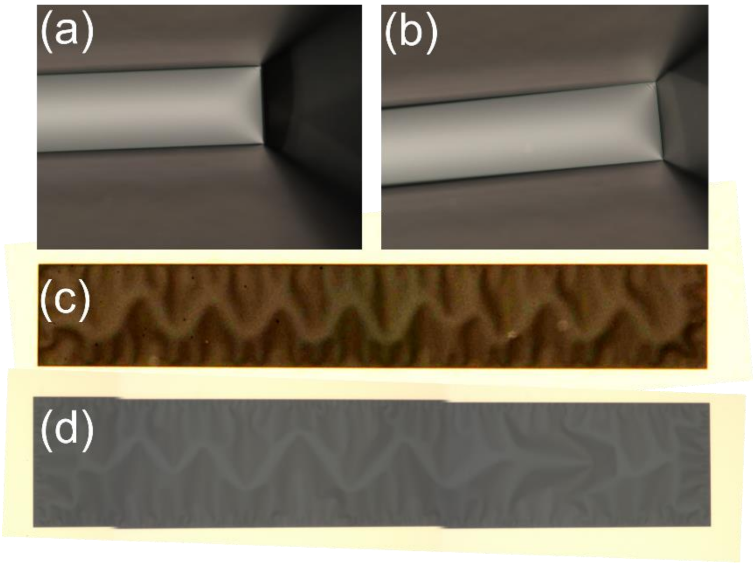

Fig S4. Ex situ optical images of 25nm-thick silicon nitride membrane mounted on a static liquid cell containing MilliQ water, (a) before and (b) after exposure to the beam. Curvature is due to the pressure exerted by the confined liquid against ambient pressure. Approximate dimensions of the membrane: 530 µm × 80 µm. Free standing silicon nitride membranes (in air) with thickness of (c) 20 nm and (c) 15 nm showing characteristic wrinkling due to residual stress [2].

References

[1] W. S. and C. J. P. W.S.M. Werner, *Simulation of Electron Spectra for Surface Analysis (SESSA) - 2.2.0*. Gaithersburg, MD, USA: National Institute of Standards and

Technology (NIST), 2021.

[2] N. Bowden, S. Brittain, A. G. Evans, J. W. Hutchinson, and G. M. Whitesides, “Spontaneous formation of ordered structures in thin films of metals supported on an elastomeric polymer,” *Nature*, vol. 393, no. 6681, pp. 146–149, May 1998, doi: 10.1038/30193.
